# A Study of *Mycobacterium tuberculosis* Detection Using Different Neural Networks in Autopsy Specimens

**DOI:** 10.3390/diagnostics13132230

**Published:** 2023-06-30

**Authors:** Joong Lee, Junghye Lee

**Affiliations:** 1 Institute of AI and Big Data in Medicine, Yonsei University Wonju College of Medicine, Wonju 26426, Republic of Korea; ljfirst@hanmail.net; 2 Postmortem Investigation Division, Department of Forensic Medicine, National Forensic Service, Wonju 26460, Republic of Korea

**Keywords:** acid-fast stain, automatic bacillus identification, computer-aided diagnosis (CAD), low resolution microscopic slide image, *Mycobacterium tuberculosis*, postmortem examination

## Abstract

Tuberculosis (TB) presents a substantial health risk to autopsy staff, given its three to five times higher incidence of TB compared to clinical staff. This risk is notably accentuated in South Korea, which reported the highest TB incidence rate and the third highest TB mortality rate among OECD member countries in 2020. The standard TB diagnostic method, histopathological examination of sputum or tissue for acid-fast bacilli (AFB) using Ziehl–Neelsen staining, demands microscopic examination of slides at 1000× magnification, which is labor-intensive and time-consuming. This article proposes a computer-aided diagnosis (CAD) system designed to enhance the efficiency of TB diagnosis at magnification less than 1000×. By training nine neural networks with images taken from 30 training slides and 10 evaluation slides at 400× magnification, we evaluated their ability to detect *M. tuberculosis*. The N model achieved the highest accuracy, with 99.77% per patch and 90% per slide. We discovered that the model could aid pathologists in preliminary TB screening, thereby reducing diagnostic time. We anticipate that this research will contribute to minimizing autopsy staff’s infection risk and rapidly determining the cause of death.

## 1. Introduction

Tuberculosis (TB) is a chronic, highly infectious, airborne disease caused by the 1- to 4-μm-long rod-shaped bacterium *Mycobacterium tuberculosis*. It ranks as the 13th leading cause of death worldwide, claiming 1.49 million lives, with 86% of all TB patients residing in India (26%), China (8.5%), and Indonesia (8.4%). The World Health Organization (WHO) forecasted an additional 137,000 TB fatalities globally for 2020 and 2021 due to the detrimental effects of COVID-19 on TB control measures. Despite a continuous decline in the incidence rate, South Korea still had the highest TB incidence rate and the third highest TB mortality rate among the 38 member countries of the Organization for Economic Cooperation and Development (OECD) in 2020. With new TB cases reaching 19,933 in 2020, the situation still presents a significant challenge [1,2,3,4,5]. 

Currently, several techniques are employed to diagnose TB infection. The commonly used methods include antibacterial plain microscopic smear (Ziehl–Neelsen (ZN) stain), fluorescence microscopic smear (auramine O, auramine–rhodamine stain), molecular tests (transcription-mediated amplification, strand-displacement amplification, conventional PCR, Xpert MTB/RIF), mycobacterial culture, drug susceptibility tests, histopathologic examination, and immunologic tests (tuberculin skin test (TST), interferon-gamma releasing assay (IGRA)). The histopathological examination using Ziehl–Neelsen (ZN) staining, being the standard method, is widely used for the diagnosis of pulmonary TB due to its affordability, simplicity, and rapidity of results [6,7,8,9,10,11]. 

Autopsy plays a crucial role in both clinical and medicolegal systems by systematically examining the deceased to determine the cause of death, offering valuable medical insights, and gathering evidence for legal investigations when needed. However, autopsy personnel can face serious health risks due to potential exposure to various infectious agents, including HIV, hepatitis B and C viruses, and *Mycobacterium tuberculosis*. The *Mycobacterium tuberculosis* bacterium is known to be robust enough to survive in diverse environments for weeks to months under favorable conditions. Viable TB bacteria have been identified in the tissues of the deceased up to 36 days postmortem. In an autopsy study of 138 formalin-fixed lung tissues with histologic evidence of TB, three specimens were cultured positive for TB, suggesting the extended risk of TB infection from handling formalin-fixed tissues. TB infection can be spread via direct contact with contaminated body fluids or tissue, but it can also be disseminated by infectious aerosols, which are airborne particles approximately 1 to 5 μm in diameter that can stay suspended in the air for extended periods. Upon inhalation, these particles traverse the upper respiratory tract and reach the alveoli. Moving and manipulating a body can expel air from the lungs of an infected individual, aerosolizing the bacilli. Aerosols can also be generated by fluid aspirator hoses discharged into sinks, oscillating saws used on bone and soft tissue, and water sprayed by hoses onto tissue surfaces. In 1979, eight of 35 medical students at the University of Sydney who attended the autopsy of an immunosuppressed patient with active tuberculosis contracted the disease. Exposure during the autopsy, even as brief as 10 min, resulted in the spread of the disease. In Finland and Japan, studies have shown that pathologists who perform autopsies are more likely to contract occupational tuberculosis than those who do not perform autopsies and those who work in university departments of preventive medicine and public health. These data emphasize the need for appropriate infection prevention measures and rapid testing [12,13,14,15,16].

In the field of medical image analysis, convolutional neural networks (CNNs) have emerged as powerful tools for disease detection. CNN models excel in analyzing medical images, such as chest X-rays and mammograms, enabling the identification of lung diseases, cancers, and specific abnormalities. Given the global burden of TB and the need for rapid screening, this study focuses on leveraging deep learning networks to classify low-resolution slide images at 400× magnification. We evaluated the performance of nine deep learning models, including NASNet-A Large, aiming to provide a preliminary screening tool to assist pathologists in shortening the diagnosis time. By improving the efficiency of TB diagnosis, this research contributes to enhancing healthcare outcomes.

This study not only addresses the challenges associated with rapid TB screening at autopsy, but it also demonstrates the potential of deep learning networks in medical image analysis for disease detection. By harnessing the power of AI models, including CNNs, we aim to improve the accuracy and efficiency of TB diagnosis, ultimately leading to better healthcare practices and outcomes.

## 2. Related Works

Abdullahi Umar Ibrahim et al. cropped 178, 524, and 530 ZN slide sets into 227 × 227 × 3 patches with a 70% training and 30% testing split. They then performed transfer learning using AlexNet. As a result, they achieved accuracies of 98.15%, 98.09%, and 98.73% in experiments A, B, and C, respectively, demonstrating an analytical ability comparable to that of a pathologist [17]. Jeannette Chang et al. used 390 fluorescence microscopy slide images (92 positive, 298 negative) and achieved accuracy of 89.2% using a support vector machine (SVM) as a classifier after Otsu binarization. They used Hu moment and a histogram of oriented gradients (HOG) as feature vectors [18]. Yan Xiong et al. used 45 ZN slides (30 positive samples, 15 negative samples) cropped into 32 × 32 × 3 patches, and they applied a CNN (convolutional neural network) model to achieve 97.94% accuracy [19]. Santiago Lopez-Garnier et al. reported that they cropped 12,510 (4849 positive, 7661 negative) images of microscopic observed drug susceptibility (MODS) 2048 × 1536 into 224 × 224 × 1 patches and trained VGG16’s CNN model with them, achieving 95.76% accuracy and 94.27% sensitivity [20]. Costa Filho et al. reported that they segmented 120 ZN microscope images using RGB, HSI, YCbCr, and Lab color separation information and classified them using SVM, achieving 97.77% accuracy [21]. Yoshiaki Zaizen et al. trained the DenseNet-121 model using 40 negative slides after annotating two positive slides and tested the results with 42 patients’ slides, achieving accuracy of 86% [22]. Moumen El-Melegy et al. used the ZNSM-iDB public database (ZN stained microscopy images) to detect regions using the Faster R-CNN (faster region-based convolutional neural network) model after cropping into 400 × 400 × 3 patches. They then resized the selected region to 20 × 20 and binary classified it with five layers of CNNs, achieving accuracy of 98.4% [23,24]. Reshma SR et al. demonstrated that contour extraction, ellipse detection, and ellipse merging techniques could be used to count *M. tuberculosis* bacteria on 176 images with 91.5% accuracy [25]. Marios Zachariou et al. demonstrated that ResNet50 can detect *M. tuberculosis* with 99.74% accuracy in 230 fluorescently stained microscope slides after segmentation by Cycle-Gan and classification of the extracted regions using ResNet, DenseNet, and SqueezeNet [26].

## 3. Materials and Methods

To diagnose tuberculosis, sputum or tissue is stained with Ziehl–Neelsen staining and examined under a microscope: the cells appear blue and complex in shape, while the waxy lipids in the cell wall of the bacillus appear red. The South Korean TB diagnosis guidelines suggest that the slides should be examined at a 1000× magnification, which is not an easy task even for pathologists [27]. In a microscopic examination, *Mycobacterium tuberculosis* is often difficult to identify due to its small size and irregular shape. The conditions of the tissue specimen and various artifacts, such as low contrast background, variations in the degree of staining, and tissue folding, can also make the bacillus even more difficult to identify. In addition, a large number of slides or small number of bacilli on a slide can require a long time to examine, which can lead to misdiagnosis due to the fatigue of the reading pathologist. Therefore, there have been requests and efforts to expedite reading with the help of artificial intelligence assistants. Recently, several models of CNNs, a new and powerful form of deep learning, have been used to detect areas of TB bacteria. Since equipment that digitizes slides at 1000× magnification is expensive, equipment that digitizes at 200/400× magnification is commonly used in practice. The ability to read at lower magnifications than the prescribed 1000× would be a major advantage in terms of applicability and time. To the authors’ knowledge, no successful studies of automated reading at lower resolutions have been published so far. The authors investigated techniques for effective detection of *Mycobacterium tuberculosis* on low-resolution digitized slide images. As described in Section 3.2, we utilized various CNN and ViT (vision transformer) models to compare their performance and explore their applicability.

### 3.1. Materials

Samples from 14 TB-infected lungs and 26 normal lungs were collected during autopsy at the National Forensic Service of Korea. All specimens were fixed, sectioned, and stained with Ziehl–Neelsen stain according to laboratory regulations to be reviewed by a pathologist. All specimens were further analyzed for tuberculosis nucleic acid amplification testing (TB-PCR) were and used in the experiment only if a match was found. All slides were scanned using a digital pathology scanner (Roche Co., Basel, Switzerland, VENTANA DP 200 slide scanner). 

**Figure 1:**
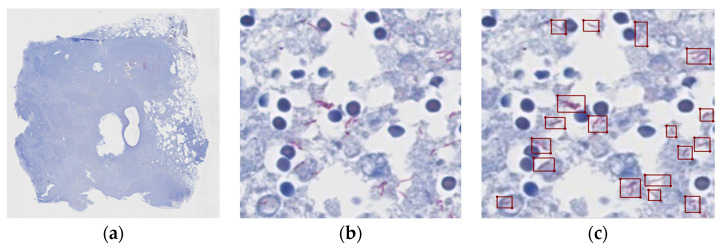
Examples of annotations. (**a**) Whole slide image of lung tissue containing *Mycobacterium tuberculosis*; Ziehl–Neelsen staining, scanning magnification view; (**b**) cropped patch image of (**a**), 400×; (**c**) annotated short, rod-shaped bacilli.

Patch-based dataset construction was performed by creating a 224 × 224 × 3 patch from an image scanned at 400× magnification, excluding the background area and including the actual tissue area. Nine of 14 positive slide samples were labeled as simple true/false by the pathologist, identifying which of the patches contained *M. tuberculosis*. Of the 351,875 positive spots, only 47,017 actually contained TB bacteria. From a total of 3.1 million negative spots, the same number of negative spots as the positive samples were randomly selected to form the complete dataset (98,034). Figure 1 shows a patch of a positive slide and the location of the tubercle bacilli.

### 3.2. Neural Network Model

CNN models are popular because they automatically extract features using a convolution layer; reduce computational complexity using a pooling layer; introduce non-linearity into the model using activation functions, such as ReLUs (rectified linear units); and improve feature extraction overall, making them more effective at learning complex patterns in large datasets than machine learning methods, such as logistic regression or decision trees. In facial recognition, for example, the Facebook team used a CNN called DeepFace to perform human-level face recognition. Andre Esteva et al. demonstrated dermatologist-level classification performance in skin cancer detection using a CNN called Inception-v3 on a large dataset of skin cancer images. Gulshan et al. outperformed the average human ophthalmologist at detecting diabetic retinopathy using Inception-v3. Pranav Rajpurkar et al. demonstrated radiologist-level performance in detecting 14 diseases from chest X-rays using a 121-layer CNN called CheXNet [28,29,30,31]. 

This section provides a brief overview of the deep learning network models used in the comparison, as shown in Table 1.

#### 3.2.1. ResNet50

**Figure 2:**
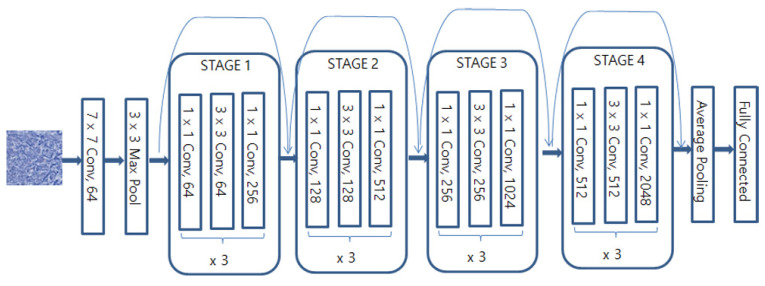
The architecture of the Resnet50 model.

Kaiming He and colleagues at Microsoft encountered a problem with the gradient disappearing as the network depth increased. To address this issue, they utilized residual learning with skip connections, which enabled deep learning to occur effectively [32]. The structure is depicted in Figure 2.

#### 3.2.2. Inception v3

**Figure 3:**
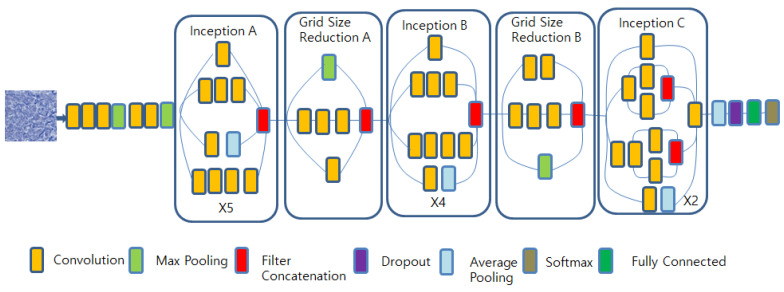
The architecture of the Inception V3 model.

Szegedy et al. created a method to learn multi-level features more effectively. This goal was accomplished by employing modules with parallel convolutions of varying sizes, which minimized computation while also addressing the problem of overfitting [33]. The structure is shown in Figure 3.

#### 3.2.3. Xception

**Figure 4:**
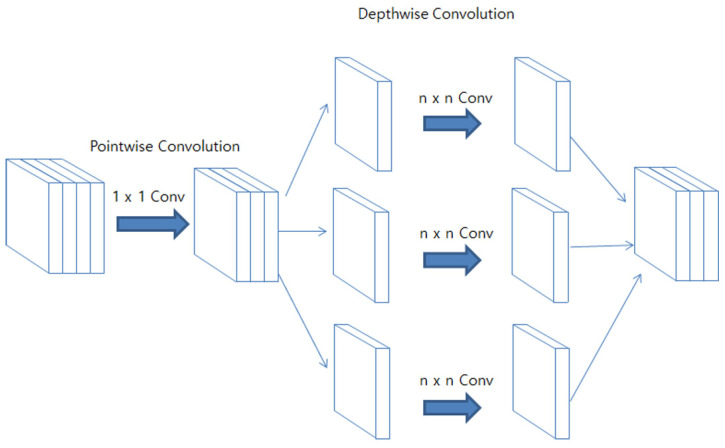
The architecture of the Xception.

The Xception architecture, an advanced form of the Inception module, effectively separates pointwise and depthwise convolutions, as illustrated in Figure 4. This arrangement allows for independent computation of cross-channel and spatial correlations. Consequently, the network can learn spatial and channel-specific features separately, boosting the efficiency of image representation learning [34]. 

#### 3.2.4. DenseNet

**Figure 5:**
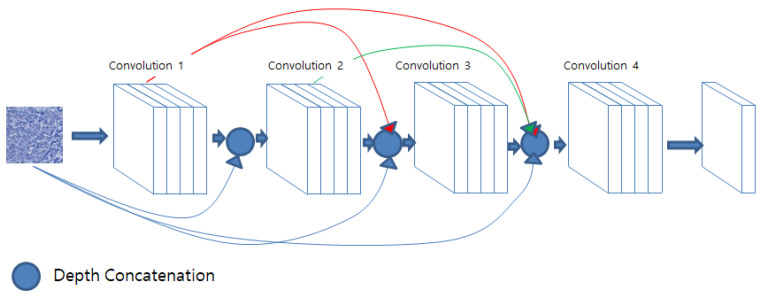
The architecture of the DenseNet.

DenseNet (densely connected convolutional networks), proposed by Huang et al., is a network structure that connects each layer’s feature map to the feature maps of all subsequent layers, as illustrated in Figure 5. This connection pattern enhances feature propagation and improves parameter efficiency [35]. 

#### 3.2.5. EfficientNet

**Figure 6:**
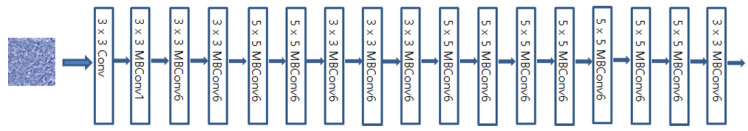
The architecture of EfficientNet.

Introduced by Tan and Le, EfficientNet systematically explores model scaling (in terms of depth, width, and resolution) as a way to achieve maximal efficiency with limited resources. The model selects efficient combinations to deliver better performance, even with smaller models [36]. The structure is shown in Figure 6.

#### 3.2.6. RegNet

Radosavovic et al. proposed RegNet, a class of models that utilize a simple design space to represent regularized network designs. The model aims to find regular (i.e., evenly distributed) networks that exhibit a simple and predictable structure while still delivering high performance [37]. 

#### 3.2.7. NASNet

NASNet is a model discovered through the neural architecture search (NAS), a method proposed by Zoph et al. NAS automates the design of neural networks. In contrast to models such as ResNet and Inception, which are manually designed by human engineers who specify and stack blocks to build the models, NASNet’s architecture consists of convolutional blocks that were automatically generated. The process of discovery used reinforcement learning and RNNs to navigate the space of possible architectures, leading to the final structure of NASNet [38].

#### 3.2.8. Vision Transformer (ViT)

Dosovitsky et al. proposed ViT, which applies transformers from the realm of natural language processing (NLP) to image classification. This model treats patches on an image as sequence elements, demonstrating that the scalability of transformers can also be leveraged for image classification tasks. This approach can deliver better results than CNN architectures and requires fewer computational resources for model training [39].

#### 3.2.9. Swin Transformer

Proposed by Liu et al., the Swin transformer is a transformer variant specifically designed for vision tasks. Like vision transformer (ViT), it breaks an image into non-overlapping patches, but it also incorporates a moving window mechanism to capture both local and global information. The Swin transformer has achieved state-of-the-art results for several vision benchmarks, including image classification, object detection, and semantic segmentation [40].

### 3.3. Experiments

In this study, we employed seven CNN architectures and two vision transformer models, as detailed in Table 1, with the aim of classifying slide images into two categories: detection and non-detection of *Mycobacterium tuberculosis*. All models were set with a mini-batch size of 24, except for NasNet, which utilized a size of 12. We adopted a learning rate of 0.001 for all models, with the exception of the two Vit-series models, which used a rate of 0.000001. The Adam optimization algorithm was implemented as the optimizer across all models. Additionally, we set the epoch count at 20 and applied an L2 regularizer with a value of 0.0001. The training process for all models was conducted utilizing the cosine annealing scheduler.

The study used RGB color images, with their sizes adjusted based on the model in use. For instance, the Inception v3, Xception, and NASNet models were supplied with images sized 299 × 299, although NASNet usually recommends a larger size. All other models were provided with images sized 224 × 224.

We applied various data augmentation techniques to the training set images, aiming to enhance model generalization and reduce overfitting risk. These techniques encompassed rotation within a range of −5 to 5 degrees, scaling from −10 to 10%, vertical and horizontal flipping, and contrast adjustment up to 10%. Additional methods included brightness adjustment, cutout augmentation, and mixup augmentation. Cutout augmentation, which erases random square sections of the input image, encourages the network to interpret more context for each pixel during training. Mixup augmentation creates synthetic examples in the input space through linear interpolation between randomly paired training examples, smoothing the model’s decision boundaries. This process reduces the overfitting likelihood and enhances the model’s generalization capabilities. For mixup augmentation, we used a mix ratio of 0.25 and an alpha value of 0.2 [41,42]. 

To preclude any overlap or bias between the training and test datasets and to effectively evaluate model performance, we implemented K-fold cross-validation (K = 5). The entire dataset was partitioned into five mutually exclusive subsets of equal size. Each subset served as a test set once, with the remaining four subsets used for training. This process enabled us to train and test on every dataset, and the average predictive performance is often used as an indicator of the model’s generalization ability. We designated 10% of the training data as the validation set and assessed the test set using the model that had the best performance during the validation phase [43]. 

To evaluate the performance of the proposed methodology, we employed the following performance metrics: accuracy, precision, sensitivity, and F1-score, which are defined in Equations (1)–(4), respectively [44].

Accuracy is the ratio of correct predictions to the total data.

(1) Accuracy=TP+TN/(TP+TN+FP+FN)

Precision is the ratio of true positive predictions to all positive predictions.

(2) Precision=TP/(TP+FP)

Sensitivity, also known as recall, is the ratio of true positives that are correctly predicted to be positive.

(3) Sensitivity=TP/(TP+FN)

F1 score is the harmonic mean of precision and sensitivity.

(4) $$F1\;\mathrm{score}=\frac{2\times \left(Precision\times Sensitivity\right)}{\left(Precision+Sensitivity\right)}$$

## 4. Results

**Figure 7:**
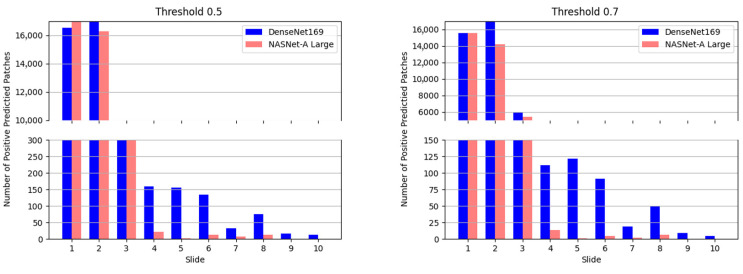
Number of positive patches per slide at two different thresholds. **Left**: 0.5 (threshold); **Right**: 0.7 (threshold).

The results of evaluating the performance of nine models using pathologist-labeled patches from 30 slides are summarized in Table 2. Notably, NASNet-A Large achieved the highest accuracy of 99.777%. We further assessed the models on a slide-by-slide basis using five negative and five positive slides, which were labeled by pathologists at the slide level rather than on a patch-by-patch basis. The evaluation was performed using the average ensemble of the five-fold models of NASNet-A Large and Densenet169 each, and the results are presented in Figure 7.

When considering only informative negative slides, the patch detection accuracy reached 99.978% and 99.820% for the NASNet-A Large and Densenet169 models, respectively. Since the slides were not labeled at the patch level, we relied on the number of positive patches for evaluation. The classification accuracy of NASNet-A Large for determining whether slides were positive or negative was 90%, while the Densenet169 model achieved 100% accuracy.

Figure 7 shows the results for a threshold of 0.5 (left) and for a threshold of 0.7 (right). Slides numbered 1 to 5 correspond to positive slides, while slides 6 to 10 represent negative slides. As depicted in Figure 7, DenseNet169 produced more estimated positives on the positive slides compared to the number of estimated positive patches on the negative slides. Increasing the threshold led to a decrease in the number of positive patches, but the number of estimated positives on the positive slides remained higher than that on the negative slides.

For the thresholds used in the experiment (0.5, 0.6, 0.7, 0.8, and 0.9), the number of false positives on the perfectly positive slide was higher than the number of false negatives. On the other hand, NASNet-A Large demonstrated that one of the positive slides had fewer false positives than the negative slide, and this error rate remained consistent regardless of the threshold. Thus, the threshold did not have a significant impact on the performance.

**Figure 8:**
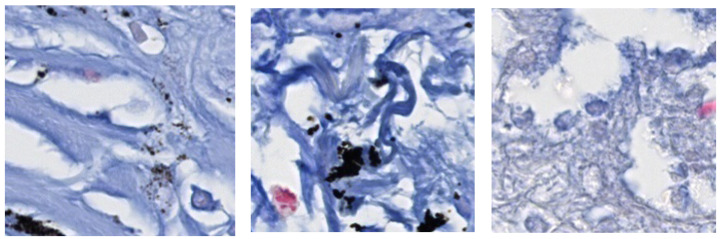
False positive patches.

Some of the presumed positive patches that were actually negative are shown in Figure 8. These errors included the appearance of clusters of *M. tuberculosis* bacteria despite the absence of *M. tuberculosis* bacteria, blurred foci of *M. tuberculosis* bacteria, and poor post-stain washing of *M. tuberculosis* bacteria.

**Figure 9:**
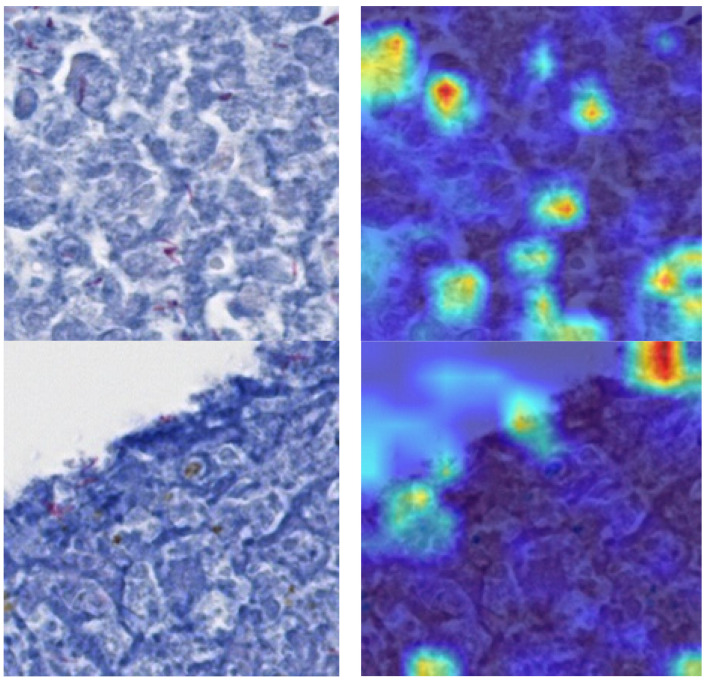
(**Left**): input image; (**right**): heat map superimposed on left image.

We also utilized gradient-weighted class activation mapping (Grad-CAM) to pinpoint on what exactly the network was focusing. We calculated the gradient of a class’s score with respect to the feature map of the penultimate convolutional layer, then globally averaged this gradient to generate the weights, and finally created the weighted combination of the activation map. If the training has been successful, the activation map will highlight the presence of *M. tuberculosis* in the patch. The degree of activation is represented by a jet color map, where blue signifies the lowest activation and red the highest. As depicted in Figure 9, there was a high level of activation for *M. tuberculosis*.

## 5. Discussion

In this study, we evaluated the performance of seven unique convolutional neural network (CNN) architectures and two vision transformer models in classifying *M. tuberculosis* slide images into two categories: detection and non-detection. This study represents a novel application of deep learning networks to the task of identifying *M. tuberculosis* on low-resolution slide scans, an approach that shows potential for revolutionizing TB diagnosis.

Among the models tested, NASNet-A Large exhibited exceptional performance, attaining accuracy of 99.777%. This superior result implies that this model could potentially provide invaluable assistance to pathologists. Additionally, our innovative incorporation of Grad-CAM to interpret the training process of the CNN architecture is a noteworthy contribution to the field. The heat map visualizations generated by Grad-CAM accurately emphasized the regions within each image patch that contained TB bacteria during training. This process provides a compelling possibility of the enhanced detection of small area lesions.

Regarding performance, the ensemble deep learning networks of Densen169 and NASNET exhibited impressive potential, with Densen169 achieving 100% positive and 100% negative separation on a slide-by-slide basis. NASNET was able to assist pathologists in making accurate judgements by requiring the examination of only a few dozen positive patches. This model could potentially increase the efficiency of TB diagnosis significantly.

**Figure 10:**
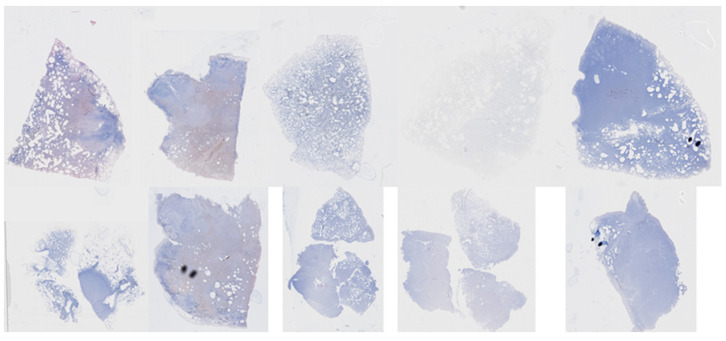
Top: negative slide images; bottom: positive slide images.

Nevertheless, it is important to acknowledge the limitations of our current approach. Despite the high per-slide accuracy levels of 90% (NASNet-A Large) and 100% (Densenet169), the limited number of slides and the narrow threshold distinguishing negative and positive cases prevent broader generalization. Moreover, upon reviewing the 40 slides represented in Figure 10, the need for an increased number of training images for improved generalization became evident. Some slides were excessively thin, displayed faint images, or showed varying staining degrees, pointing to a more diverse range of real-world cases. To create a robust model capable of effectively handling these varied cases, extensive training with a larger and more diverse set of slide images is indispensable. 

In our future work, we aim to collect additional slide samples to improve the model’s performance and applicability to low-resolution slides at magnification of 200×.

## 6. Conclusions

Tuberculosis (TB) remains a serious global health issue, claiming approximately 1.49 million lives annually. The risk is high even in countries with a high incidence of TB, such as South Korea, particularly during autopsies. Standard TB testing involves a labor-intensive and time-consuming examination of slides at 1000× magnification following Ziehl–Neelsen staining.

In response to this challenge, there have been attempts to develop automated TB sputum tests using high-resolution microscopic images. However, these techniques often prove inadequate in settings where slides can only be digitized using low-resolution scanners. In our study, we evaluated the performance of nine deep learning networks in classifying low-resolution slide images at 400× magnification. The NASNet-A Large model achieved remarkable accuracy of 99.777%, demonstrating its potential for automated testing.

Furthermore, Densen169 showed 100% accuracy on a slide-by-slide basis, and NASNET enabled pathologists to make accurate judgements swiftly, as they only needed to inspect a few dozen positive patches. This ability demonstrates potential support for preliminary testing by pathologists to reduce diagnostic time, which could in turn enhance the efficiency of TB diagnosis and contribute to preventing TB.

Moving forward, we plan to improve the performance of our methodology by expanding the training dataset with additional samples from various hospitals and laboratories. This comprehensive dataset will facilitate the training of more generalized and robust deep networks, ultimately providing a more efficient tool for the rapid and precise diagnosis of TB. We are optimistic that our efforts will make significant strides toward faster and more accurate TB diagnosis.

## Figures and Tables

**Table 1:** Overview of the deep learning network models used in the comparison.

Model	Depth	Input Size
ResNet50	50 layers (Convolutional + Fully Connected)	224 × 224
Inception v3	48 layers (Inception modules + Fully Connected)	299 × 299
Xception	71 layers (Convolutional + Separable Convolutions)	299 × 299
DenseNet169	169 layers (Convolutional + Pooling + Batch Normalization)	224 × 224
EfficientNet-B0	Varied due to compound scaling method	224 × 224
RegNetY-064	64 stages (Sequence of Convolutional layers)	224 × 224
NASNet-A Large	280 layers (Convolutional)	332 × 332
Vit_base_patch16_224	12 Transformer layers	224 × 224
Swin Transformer Small	110 layers (due to hierarchical structure)	224 × 224

**Table 2:** Results of evaluation for neural networks. The best results are highlighted in bold.

Model	Accuracy (%)	Precision (%)	Recall (%)	F1 (%)
ResNet50	98.970 ± 0.033	98.973 ± 0.035	98.970 ± 0.033	98.970 ± 0.033
Inception v3	98.949 ± 0.049	98.952 ± 0.049	98.949 ± 0.049	98.949 ± 0.049
Xception	98.980 ± 0.105	98.981 ± 0.106	98.980 ± 0.105	98.980 ± 0.105
DenseNet169	98.980 ± 0.098	98.982 ± 0.098	98.980 ± 0.098	98.980 ± 0.098
EfficientNet-B0	98.928 ± 0.031	98.931 ± 0.030	98.928 ± 0.031	98.928 ± 0.031
RegNetY-064	98.932 ± 0.078	98.935 ± 0.079	98.932 ± 0.078	98.932 ± 0.078
NASNet-A Large	**99.777** ± 0.0231	**99.728** ± 0.0356	**99.771** ± 0.0175	**99.749** ± 0.0260
Vit_base_patch16_224	98.667 ± 0.055	98.668 ± 0.054	98.667 ± 0.055	98.667 ± 0.055
Swin Transformer Small	98.918 ± 0.0407	98.920 ± 0.0410	98.918 ± 0.0407	98.918 ± 0.0407
ResNet50	98.970 ± 0.033	98.973 ± 0.035	98.970 ± 0.033	98.970 ± 0.033

## Data Availability

Data are available from the first author and the corresponding author upon request.
